# Oncolytic viruses enhance CAR-T activity for the treatment of pediatric diffuse midline gliomas

**DOI:** 10.1016/j.omton.2026.201255

**Published:** 2026-06-16

**Authors:** Kyung-Don Kang, Zhuo Zhang, Joshua D. Bernstock, Gregory K. Friedman

**Affiliations:** 1 Division of Pediatrics, The University of Texas MD Anderson Cancer Center, Houston, TX, USA; 2 Department of Neurosurgery, Brigham and Women’s Hospital, Harvard Medical School, Boston, MA, USA

## Main text

Diffuse midline glioma (DMG) remains one of the most lethal pediatric brain tumors, with a median survival of less than one year despite aggressive treatment.^1^ DMGs arise predominantly within midline structures of the central nervous system, most commonly the pons, and tend to occur during defined developmental windows that may confer distinct epigenetic and microenvironmental vulnerabilities.^2^ Despite substantial advances in the molecular understanding of these tumors, radiotherapy remains the primary established upfront treatment and provides only transient clinical benefit. However, ONC201, recently approved by the FDA in 2025 for recurrent H3K27M-mutant DMG, is increasingly being explored in frontline treatment settings.^3^ Nonetheless, the development of effective therapeutic strategies for DMG remains an urgent unmet clinical need.

Multiple novel therapeutic strategies are currently under clinical investigation for DMG, including chimeric antigen receptor (CAR) T cell therapy and oncolytic viruses (OVs).^4^^,^^5^ CAR T cell therapy has demonstrated remarkable clinical efficacy in hematologic malignancies; however, durable responses in solid tumors, including DMG, remain limited by antigen heterogeneity, T cell exhaustion, impaired trafficking, and profoundly immunosuppressive tumor microenvironments (TMEs). In parallel, OVs are increasingly recognized not only as direct tumor-lytic agents but also as potent immune modulators capable of reshaping the TME through inflammatory priming and cytokine production.^6^^,^^7^ Consequently, OVs represent an attractive combinatorial partner for CAR T cell therapy in DMG. Nevertheless, several fundamental biological questions remain regarding OV-CAR T therapy. First, whether OVs directly impair CAR T cell viability, and second, what is the mechanistic basis underlying OV-mediated enhancement of CAR T cell cytotoxicity?

In the previous issue of *Molecular Therapy Oncology*, Vazaios et al.^8^ investigated the biological compatibility and therapeutic potential of combining CAR T cells targeting B7-H3 and GD2 with OVs, Goravir (adenovirus), and R124 (orthoreovirus), in DMG models. Importantly, the investigators demonstrated that the CAR T cells were relatively resistant to OV-mediated toxicity compared with untransduced donor T cells, supporting the feasibility of combining the OVs with the CAR T cells while also emphasizing the importance of treatment timing and viral dosing.

To further define OV-induced cellular responses, the authors performed bulk RNA sequencing analyses following viral infection. Interestingly, Goravir predominantly suppressed DMG cell cycle and proliferative pathways, whereas R124 preferentially induced interferon-stimulated and inflammatory gene programs. These findings suggest that distinct OV platforms may generate fundamentally different immune and transcriptional landscapes within the TME.

The authors next evaluated whether OV pre-infection alters CAR T cell-mediated anti-tumor activity. Intriguingly, OV pre-treatment enhanced CAR T cell cytotoxicity in several DMG models, whereas other DMG models failed to demonstrate significant therapeutic enhancement. Accordingly, the investigators classified tumors into “responder” and “non-responder” groups based on the degree of OV-mediated augmentation of CAR T cell killing. This distinction represents one of the most important conceptually important findings of the study, suggesting that tumor-intrinsic immune programs may critically influence susceptibility to OV-mediated immune priming.

Mechanistically, enhanced anti-tumor responses following OV pre-infection correlated with increased cytokine and chemokine release by both B7-H3 and GD2 CAR T cells. Notably, R124 induced broader cytokine alterations in responder tumors compared with Goravir, further supporting the superior immunostimulatory properties of R124. These observations suggest that successful OV-CAR T combinational therapy may depend less on direct viral oncolysis and more on the ability of OVs to induce inflammatory remodeling of the TME.

Finally, the investigators examined phenotypic changes within CD4 and CD8 CAR T cell subsets following OV pre-infection. OV-responder DMGs promoted substantial phenotypic remodeling characterized by increased expression of TIM-3 and TIGIT, as well as enhanced CD56 and CCR7 expression associated with cytotoxic and memory-related programs. These findings suggest that OV-mediated immune priming may reshape CAR T cell functional states in addition to enhancing direct tumor cell killing.

Based on these findings, differential responsiveness to OV-mediated immune priming may help explain the substantial biological heterogeneity observed in DMG. Defining these immune states may ultimately enable more rational selection of single OV platforms, multi-OV combinations, or OV-CAR T cell therapeutic strategies tailored to individual patients.

In conclusion, this study supports a growing paradigm in which OVs function not only as tumor-lytic agents but also as potent immune modulators capable of enhancing CAR T cell activity in DMG. Importantly, the identification of responder and non-responder phenotypes raises the possibility that future OV-CAR T strategies may ultimately require biomarker-driven patient stratification based on tumor-specific immune states and susceptibility to inflammatory remodeling. Further studies integrating spatial and single-cell immune profiling may help define the molecular determinants governing responsiveness to combinatorial immunovirotherapy in pediatric brain tumors.

## Declaration of interests

J.D.B. has an equity position in Treovir Inc., an oncolytic oHSV company. J.D.B. is also a co-founder of Centile Bioscience and Oncolight serves on the boards of NeuroX1, QV Bioelectronics, Upfront Dx, and Synaptive Medical. He also has a provisional patent (application number 63/273,577) entitled “Methods and formulations related to the intrathecal delivery of oncolytic viruses”. G.K.F. holds an equity interest in Synaptive Medical, Inc. and a patent for methods and formulations related to the intrathecal delivery of oncolytic virus. He received prior funding to his institution from Pfizer, Eisai, and E.R. Squibb & Sons for research unrelated to the content of this manuscript.
